# Osteoporosis, an Inevitable Circumstance of Chronic Kidney Disease: A Systematic Review

**DOI:** 10.7759/cureus.18488

**Published:** 2021-10-05

**Authors:** Nishat Tasnim, Priyata Dutta, Jannatun Nayeem, Parisha Masud, Afsana Ferdousi, Ammy S Ghosh, Maksuda Hossain, Sultana Rajia, Khadija T Kubra, Md Sakibuzzaman, Asma T Khan

**Affiliations:** 1 Internal Medicine, Sacramento Veterans Affairs Medical Center, Mather, USA; 2 Internal Medicine, Mymensingh Medical College, Mymensingh, BGD; 3 Internal Medicine, Cumilla Medical College and Hospital, Cumilla, BGD; 4 Internal Medicine, Columbia University Medical Center, New York, USA; 5 Internal Medicine, California Institute of Behavioral Neurosciences & Psychology, Fairfield, USA; 6 Internal Medicine, Institute of Applied Health Sciences, Chittagong, BGD; 7 Biodesign Institute Center for Immunotherapy, Vaccines and Virotherapy, Arizona State University, Tempe, USA; 8 Internal Medicine, Sher-E Bangla Medical College, Barishal, BGD; 9 Internal Medicine, Bangladesh Medical College and Hospital, Dhaka, BGD; 10 Internal Medicine, University of Mississippi Medical Center, Jackson, USA; 11 Experimental Pathology (Cancer Biology), Mayo Clinic, Rochester, USA; 12 Internal Medicine, Sir Salimullah Medical College, Dhaka, BGD; 13 Neuroscience, California Institute of Behavioral Neurosciences & Psychology, Fairfield, USA; 14 Division of Research & Academic Affairs, Larkin Community Hospital, South Miami, USA

**Keywords:** abnormal bone turnover, fibroblast growth factor, vitamin d, calcium, pth, end-stage renal disease (esrd), fracture, bone fragility, osteoporosis, ckd

## Abstract

Nowadays, chronic kidney disease (CKD) and osteoporosis have become crucial health-related issues globally. CKD-induced osteoporosis is a systemic disease characterized by the disruption of mineral, hormone, and vitamin homeostasis that elevates the likelihood of fracture. Here, we review recent studies on the association of CKD and osteoporosis. In particular, we focus on the pathogenesis of CKD-associated osteoporosis, including the homeostasis and pathways of several components such as parathyroid hormone, calcium, phosphate, vitamin D, fibroblast growth factor, and klotho, as well as abnormal bone mineralization, remodeling, and turnover. In addition, we explore the diagnostic tools and possible therapeutic approaches for the management and prevention of CKD-associated osteoporosis. Patients with CKD show higher osteoporosis prevalence, greater fracture rate, increased morbidity and mortality, and an elevated occurrence of hip fracture. We also rule out that increased severity of CKD is related to a more severe condition of osteoporosis. Furthermore, supplements such as calcium and vitamin D as well as lifestyle modifications such as exercise and cessation of smoking and alcohol help in fracture prevention. However, new approaches and advancements in treatment are needed to reduce the fracture risk in patients with CKD. Therefore, further collaborative multidisciplinary research is needed in this regard.

## Introduction and background

Chronic kidney disease (CKD) has become a global public health issue. CKD refers to a systemic disorder that causes kidney functions to gradually deteriorate with the glomerular filtration rate (GFR) falling below 60 mL/minute/1.73 m^2^ for three months or more [1]. The National Kidney Foundation’s Kidney Disease Outcomes Quality Initiative (KDOQI) divides CKD into several stages [2]. Table 1 summarizes these stages of CKD and their respective GFR levels [2].

**Table 1:** CKD staging. CKD: chronic kidney disease

Stages of CKD	GFR 1.73 mL/minute/1.73 m^2^	
Stage 1 (normal)	>= 90	
Stage 2 (mild deterioration)	60–89	
Stage 3A (mild-to-moderate deterioration)	45–59	
Stage 3B (moderate-to-severe deterioration)	30–44	
Stage 4 (severe deterioration)	15–29	
Stage 5 (kidney failure)	<= 15	
Stage 5D (kidney failure)	On dialysis	

CKD and mineral bone disorder (MBD) are heavily intertwined [1,3]. Over time, this condition leads to osteoporosis and end-stage renal disease (ESRD) [4].

The National Institutes of Health Consensus Development Panel defines osteoporosis as a disorder of the skeleton caused due to a reduction in bone strength, thus leading to an elevated predisposition for fractures [2]. It affects two main components of bone strength: bone quality and bone quantity. Bone quality characterizes the internal structure of the bone, including minerals, connective tissues, and trabecular and cortical compartments, and is measured using quantitative computed tomography (QCT) or high-frequency resolution tomography (HR-pQCT) [2]. Furthermore, bone quantity characterizes the level of bone resorption and is measured using dual-energy X-ray absorptiometry (DXA) [2].

CKD-induced osteoporosis is a systemic disease that can be related to the following (one or more): abnormal calcium metabolism, phosphate, vitamin D, parathyroid hormone (PTH); calcification of soft tissue; and abnormal bone turnover and mineralization [1,3]. It is linked to an elevated risk of fragility fractures, morbidity, and mortality, which implies that this condition causes a wide range of health-related problems [1,4]. Moreover, bone fragility is frequent in CKD patients compared to patients with regular kidney function [5]. Bone fracture, especially hip fracture, is nearly double in CKD patients older than 50 years compared to normal individuals [5]. This high risk of fracture significantly impairs regular activity and quality of life. Therefore, in this study, we investigate the correlation between CKD and osteoporosis along with their pathogenesis, diagnosis, management, and preventive strategies against fracture.

Here, we first describe the epidemiology and risk factors of CKD-associated osteoporosis. Then, we present a comprehensive review of the literature using appropriate keywords based on our inclusion and exclusion criteria. Subsequently, we provide an overview of the pathophysiology of CKD-induced osteoporosis and its current diagnostic, therapeutic, and preventive strategies. In addition, we shed light on the key findings of previous works associated with this topic and the possible limitations of this study. Finally, the conclusion provides a summary of our work and emphasizes how early intervention can mitigate the morbidity associated with this disease.

## Review

Epidemiology and risk factors

The incidence and prevalence of CKD have been increasing significantly worldwide. It is more prevalent among the elderly along with hypertension, diabetes mellitus, and other health-related issues [5]. CKD is estimated to affect about 8% to 16% of the global population [4]. Around 772 million individuals are diagnosed with CKD globally [2]; among them, more than 20 million individuals are affected in the United States [2]. CKD is often associated with MBD, osteoporosis, and fragility fracture [4]. For women over 65 years with GFR of <15 mL/minute/1.73 m^2^, the fracture risk has been reported to be three times higher in comparison to women with GFR of ≥60 mL/minute/1.73 m^2^ [6]. However, in men aged 40-65 years with GFR of <15 mL/minute/1.73 m^2^, fracture risk has been reported to be five times higher in comparison to men with GFR of >60 mL/minute/1.73 m^2^ [6]. Hip fracture risk is four times higher in hemodialysis patients [6]. Moreover, low serum sodium is also considered a leading risk factor that can contribute to the increased susceptibility to bone fracture in CKD patients. Neurological disturbance followed by gait abnormality in chronic hyponatremia can increase the risk of hip fracture [7]. However, individuals of older age, female sex, caucasian race, and low body mass index (BMI) are at a higher likelihood of having a fracture compared to those of younger age, male sex, African American, and high BMI [8]. Therefore, identifying these risk factors and implementing proper management can mitigate adverse outcomes.

Methodology

**Figure 1:**
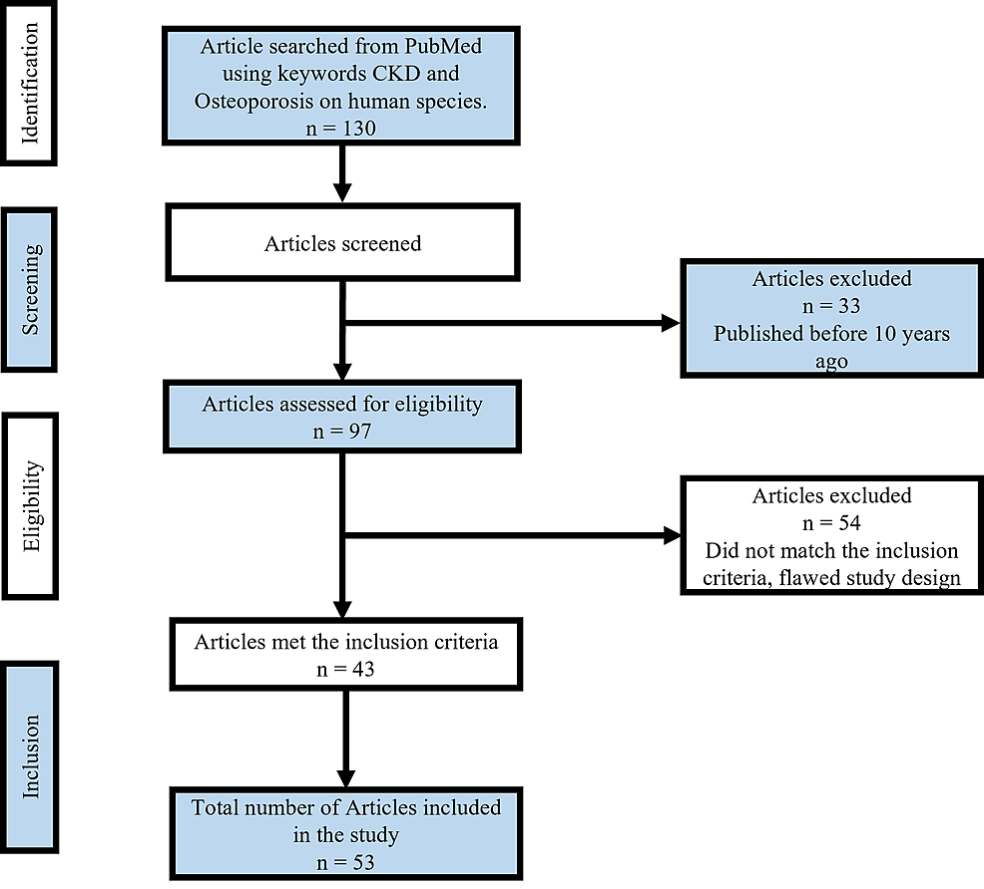
Flowchart showing the article selection procedure followed in this work. CKD: chronic kidney disease

We searched the PubMed database using appropriate keywords: “chronic kidney disease” and “osteoporosis.” The included studies were restricted to studies published from 2011 to 2021, on human subjects, written in English, and with full text available. The screening process was in consonance with the Preferred Reporting Items for Systematic Reviews and Meta-Analyses (PRISMA) protocol [9], as shown in Figure 1. We reviewed 130 studies carefully and checked the references thoroughly to avoid duplication. We ruled out studies with a flawed design and a different outcome that did not match our inclusion criteria. After reviewing and refining the results of the search, 43 articles were selected. With another 10 relevant articles, a total of 53 articles were included in our study.

Discussion

Basic Aspects of Pathogenesis of Chronic Kidney Disease-Induced Osteoporosis

Calcium, phosphate, vitamin D, and PTH play key roles in bone formation and mineralization by maintaining normal bone homeostasis. Regulation of mineral and bone metabolism relies on the regulation of calcium and phosphate, which depends on the function of several components, such as PTH, vitamin D, calcitonin, fibroblast growth factor-23 (FGF-23), and klotho. These components maintain serum calcium and phosphate levels within the normal ranges by modulating the absorption, excretion, and storage of both elements. The parathyroid gland, kidney, and intestine are the organs involved in this mechanism. CKD alters this normal regulation and increases the risk of fracture. The investigation of their influence on bone helps in understanding the mechanism of CKD-associated osteoporosis [10,11].

Abnormal calcium, phosphate, vitamin D, and parathyroid homeostasis: In CKD patients, kidneys retain phosphate, leading to decreased serum calcium and vitamin D. However, serum calcium level is not reduced until the GFR drops below 20 mL/minute/1.73 m^2^ [5,12]. The calcium-sensing receptor (CaSR), a receptor on the parathyroid gland, detects this reduction in calcium level, thus stimulating PTH release from the parathyroid gland [9]. In CKD patients, hyperactivated PTH alters bone mechanical properties by reducing bone mass and abnormal remodeling of bones to restore serum calcium to its normal range [13]. CaSR expression may be reduced by overgrowth of the parathyroid gland, and due to this reduction, even hypercalcemia cannot suppress PTH secretion. Eventually, elevated PTH increases the risk of osteoporotic fracture [9].

As CKD damages functioning nephrons, the excretion of phosphate is hampered, leading to its retention in the body. Therefore, FGF-23 and PTH aid in the modification of this mechanism by decreasing phosphate reabsorption in the persisting nephrons [14,15]. Therefore, serum phosphate usually remains normal in the early stages of CKD and until GFR is 20 mL/minute/1.73 m^2^ [12]. Subsequently, PTH becomes the main adaptive mechanism for phosphate hemostasis as the effects of FGF-23 on phosphate excretion become limited due to klotho deficiency in the renal tubule [15]. Hyperphosphatemia in advanced CKD causes hyperplasia of the parathyroid gland and stimulates the production and secretion of PTH [14]. However, hyperphosphatemia reduces vitamin D activity in the kidney and develops calcitriol (active metabolite of vitamin D) insufficiency that leads to defective bone mineralization and fragility, whereas hyperphosphatemia stimulates FGF-23 production in the bones [14,16,17].

Vitamin D, along with calcium and phosphate metabolism, plays an important role in bone formation and mineralization [18]. In patients with CKD, vitamin D production is decreased due to renal failure. Therefore, in vitamin D-deficient patients (<15 nmol/L), bone formation and trabecular mineralization are reduced as calcium level is also decreased [18]. This condition is compensated by increased PTH (secondary hyperparathyroidism [SHPT]) that reconstructs 25-hydroxyvitamin D into the active form of vitamin D [19,20]. Furthermore, deficiency of vitamin D aggravates SHPT and elevates the risk of proteinuria, bone turnover and mineralization abnormality, bone loss, and mortality [20,21]. However, proteinuria can also cause vitamin D deficiency due to a decrease in calcium-binding proteins [18,22]. Nevertheless, the severity of CKD is strongly associated with inflammatory mediators and insufficiency of vitamin D. With the progression of CKD, more inflammatory factors are released, phosphate levels increase, and calcium levels decrease. Increased phosphate level activates the NF-κB pathway and leads to an increased level of tumor necrosis factor-alpha (TNF-α), thus causing overactivation of the inflammatory system [23].

PTH is a crucial representative biomarker for bone fragility [23]. It usually remains normal until GFR is 45 mL/minute/1.73 m^2^, and its elevation reduces GFR [12,24,25]. Higher PTH is an index of lower bone mineral density. Osteoporotic patients have higher PTH levels than osteopenic patients [13]. With the advancement of CKD, the prevalence of SHPT rises [12,25]. Ultimately, SHPT leads to abnormal bone remodeling, turnover, and fracture. Causes of SHPT include phosphate retention, calcium and vitamin D reduction, and FGF-23 elevation, as well as decreased expression of CaSR, vitamin D receptor, FGF-23 receptor, and klotho in the parathyroid gland. Moreover, the expression of the parathyroid receptor is reduced by uremic toxins, for example, p-cresyl sulfate and indoxyl which increases cyclic adenosine 3', 5' monophosphate (cAMP) synthesis in the osteoblast [9,13,26]. This cAMP influences skeletal resistance to PTH and eventually osteoporotic fracture [9].

Fibroblast growth factor and klotho: FGF is a polypeptide growth factor [27,28] and a phosphotrophic hormone produced by osteoblasts and osteocytes [28-30]. Its biosynthesis is positively regulated by high plasma phosphate and PTH. Bioactive FGF-23 acts on the kidney and parathyroid gland and maintains the homeostasis of its regulators via binding to FGF receptor-1 (FGFR1) [27,28]. However, the interaction between FGF-23 and FGFR1 is weak; hence, the coreceptor klotho increases FGF-23 affinity to its receptor [27]. Klotho is a pleiotropic protein involved in the inhibition of fibrosis, cellular aging, and apoptosis [27,30]. FGF-23, along with klotho, plays a vital role in mineral and hormone homeostasis, bone health, and mineralization [29]. In the kidney, the interaction between FGF-23 and FGFR1/klotho complex results in decreased phosphate reabsorption by internalization and degradation of the sodium-phosphate cotransporter. It suppresses the formation of vitamin D by reducing the expression of 1α hydroxylase and increases calcium reabsorption in the distal tubule by upregulating calcium-selective channel proteins [27,28,30]. FGF-23 also decreases PTH secretion by inhibiting its gene expression [27,28]. CKD causes alteration in mineral metabolism, augmented by alteration in FGF-23 and klotho levels [31]. The first metabolic derangement of CKD is phosphate retention; to reduce phosphate burden, bone increases FGF-23. However, in the early stages of CKD, downregulation of klotho occurs due to loss of viable tissue, albuminuria, and activation of the Wnt/β-Catenin signaling pathway; therefore, elevated FGF-23 cannot function, leading to FGF-23 resistance [27,28,31]. As CKD progresses, the plasma concentration of FGF-23 increases, klotho decreases, phosphate accumulates, urinary calcium wasting is promoted, and vitamin D synthesis is suppressed due to renal resistance to FGF-23 [27,31]. The resultant mineral disruption causes SHPT and osteoporosis.

Abnormal bone mineralization, remodeling, and turnover: The quality of the bone depends on bone mineralization, remodeling, turnover, microdamage, and cortical-trabecular microarchitecture [2]. Bone strength depends on cortical and trabecular bone; however, bone (trabecular) contributes higher, encompassing about 80% of the framework [2,4]. Changes in the trabecular and cortical bone are more pronounced in CKD patients compared to the general population. Moreover, cortical bone loss occurs more rapidly than trabecular bone loss as elevated PTH level has an anabolic outcome on the bone (trabecular) whereas a catabolic outcome on (bone) cortical [2]. Hence, in postmenopausal women without CKD, trabecular bone damage occurs more rapidly. PTH also causes cortical bone loss by increasing porosity and decreasing thickness [2]. Nonetheless, overall bone quality is degraded by the accumulation of glycation products and increased oxidative stress. Glycation products loosen collagen networks, and oxidative stress forms microcracks and eventually bone fracture [32-34].

**Figure 2:**
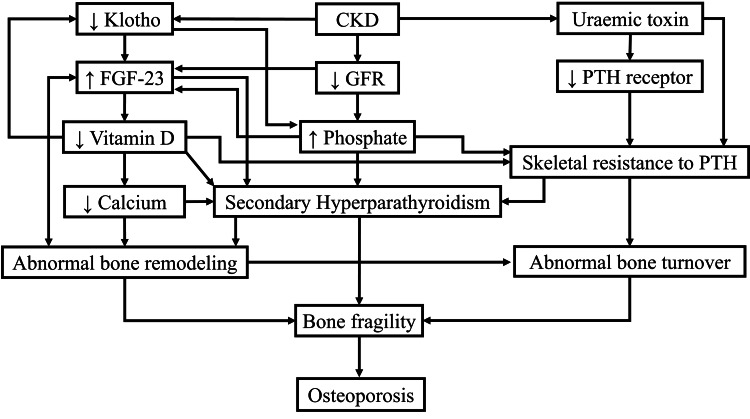
A summary of mechanisms showing the effects of CKD on osteoporosis. CKD: chronic kidney disease; FGF-23: fibroblast growth factor-23; GFR: glomerular filtration rate; PTH: parathyroid hormone

In short, due to abnormal homeostasis in CKD, phosphate and FGF-23 are elevated and klotho, vitamin D, and calcium levels are reduced which leads to SHPT. CKD also stimulates uremic toxins, which causes skeletal resistance to PTH [5,9]. Together, SHPT and skeletal resistance to PTH eventually result in abnormal bone remodeling, turnover, bone fragility, and finally osteoporosis [9], as shown in Figure 2.

Diagnostic Approaches

Noninvasive imaging techniques: Skeletal imaging is used to evaluate fracture risk and skeletal abnormalities in individuals [2]. This includes DXA, QCT, HR-pQCT, and micro-MRI [33]. DXA has been used widely in day-to-day practice. Although it can measure bone quantity, it cannot measure bone quality [2]. QCT, HR-pQCT, and micro-MRI can measure the quality, solidity, geometry, three-dimensional density, microarchitecture, and structural characteristics of bones [35,36]. DXA is reported as T-score, which measures total hip and spinal bone mineral density (BMD) and is demonstrated as grams per square centimeter. Usually, the T-score is computed by the National Health and Nutrition Examination Survey 111 Reference Database [37]. Recent US practice guidelines recommend DXA every one to two years for people over 65 years of age with one or more fracture risk factors [2,38]. The Fracture Risk Assessment Tool is a risk prediction model that provides a 10-year absolute risk of osteoporotic fracture by combining 10 clinical risk factors, including age, sex, smoking, BMI, previous fracture, family history of fracture, and secondary forms of osteoporosis [4].

Invasive techniques: Bone biopsy is the gold standard procedure used for histomorphological categorization. It also helps in shedding light on histomorphometric abnormalities in patients with stage 3-5 CKD [2,39-41]. However, it is not commonly used as it is expensive, painful, time-consuming, widely unavailable, and not useful to predict fracture risk [2]. Bone turnover markers are a substitute for the measurement of bone biopsy. These are classified into two categories and are more convenient when they are at the utmost of their ranges [2,42]. Bone formation parameters include specific alkaline phosphatase, osteocalcin, and procollagen type 1 n propeptide. On the other hand, bone resorption parameters involve PTH and tartrate-resistant acid phosphatase 5b [2].

Management

Management of osteoporosis in patients with CKD has been challenging as several therapeutic agents used to treat osteoporosis can affect renal function. Treatment options for patients with stage 1-3 CKD are almost the same as patients without CKD. Treatment options for stage 4 CKD patients or those on dialysis depend on creatinine level and GFR. The most common treatment used for osteoporosis is bisphosphonates [43]. The Food and Drug Administration (FDA) recommends that patients with a creatinine clearance of >35 mL/minute can use bisphosphonates such as alendronate, risedronate, minodronic acid, ibandronate, and etidronate cautiously. However, patients who have a creatinine clearance of <35 mL/minute should not use bisphosphonates as they are cleared by the kidney [8,11,43]. Yet, the consequence of bisphosphonate treatment on BMD is undetermined [44]. However, studies have shown that risedronate significantly elevates BMD and reduces vertebral fractures in osteoporotic women without causing any adverse effect on renal function [45].

The American Society of Nephrology conducted the FREEDOM Trial which recommended denosumab [46]. This antiresorptive agent did not show any interaction between kidney function and treatment effect. Iseri et al. [47] reported that denosumab increases BMD and improves bone biomarkers. However, another trial reported that approximately 15% of patients using denosumab experience mild-to-moderate hypocalcemia, which can be avoided with adequate supplementation of vitamin D and calcium [41]. Another recommended management option is the selective estrogen receptor modulator, known as raloxifene, which is approved by the FDA [11,48]. Studies reported improved BMD by raloxifene in postmenopausal osteoporotic patients with stage 1-5 CKD without any significant adverse effects [11]. Moreover, teriparatide (an anabolic agent) and abaloparatide (a recombinant of PTH) can also be employed to manage osteoporosis in these patients [11]. Nonetheless, alfacalcidol (an active vitamin D analog) has also shown effectiveness in postmenopausal and male osteoporosis [49].

Preventive Approaches

Fracture prevention is one of the primary goals of osteoporosis management in patients with CKD. The treatments discussed above can increase bone mass and reduce the incidence of fracture [8]. However, it is vital to add supplementation to reduce the risk of fracture in these patients. Sufficient food intake, adding vitamin D, reducing phosphate intake, introducing calcimimetics, and evaluating each patient whether they need parathyroidectomy are essential factors for preventing fracture and treating these patients [35,50]. Nevertheless, avoidance of falls in the elderly and lifestyle modifications, such as exercise, cessation of smoking, and abstinence from alcohol, also help in fracture prevention [9,51,52]. Therefore, the abovementioned preventive approaches and careful application of appropriate medications after evaluating kidney function together halt the possibility and progression of fracture [53].

Key Findings from Prior Research Works

We reviewed a total of 53 articles in this work. Key findings from 12 papers are summarized in Table 2. Our analysis showed that hypocalcemia, hyperphosphatemia, hyperparathyroidism, vitamin D and klotho deficiency, increased concentration of FGF-23, and abnormal bone mineralization and turnover contribute to the development of osteoporosis in patients with CKD [5,9]. These patients show a higher percentage of osteopenia and osteoporosis and a >10‐fold higher fracture rate than those without CKD [13,35]. This leads to increased morbidity and mortality [38]. Huang et al. [1] observed a higher prevalence of hip fracture than spine; however, Chen et al. [24] reported an increased incidence of fracture anywhere in the body. An observational cohort study also established that patients with CKD who had a lower BMD also had a more significant occurrence of ESRD than those with normal BMD [53]. Prior research also indicated that DXA should be considered to guide CKD-related treatment as it provides an accurate diagnostic technique in CKD patients [4]. Finally, sufficient food intake, supplementation of calcium and vitamin D, prevention of falls, and lifestyle modification, such as exercise, termination of smoking, and abstinence from alcohol, may help in fracture prevention [9,35,50,51]. In conclusion, an integrative management proposition is needed due to the multiplexity of bone fracture in CKD patients [50].

**Table 2:** Summary of the key findings from prior studies. CKD: chronic kidney disease; BMD: bone mineral density; eGFR: estimated glomerular filtration rate; FN: femoral neck; LS: lumbar spine; ESRD: end-stage renal disease; PTH: parathyroid hormone; MBD: mineral bone disorder; IL-6: interleukin-6; CRP: C-reactive protein; TNF-α: tumor necrosis factor-alpha; DXA: dual-energy X-ray absorptiometry; SERM: selective estrogen receptor modulator; RANKL: receptor activator of nuclear factor kappa-Β ligand; ROD: renal osteodystrophy; FRAX: Fracture Risk Assessment Tool; iPTH: intact parathyroid hormone; bALP: bone alkaline phosphatase; FGF: fibroblast growth factor

Author and year of publication	Study design	Summary	Limitation	Conclusion
Hall et al., 2018 [38]	Prospective cohort	A 10-year-long prospective cohort study was performed on older male veterans at the Department of Veterans Affairs national healthcare system. Among them, 808,525 patients had CKD and 3,529,664 patients did not have CKD. Results show that 15.7% of patients with CKD experienced a fracture irrespective of age, race, and BMD, and 43.0% of them died over 5.2 years	N/A	Patients with stage 3 CKD have greater morbidity and a higher risk of mortality
Huang et al., 2020 [1]	Cross-sectional study	A total of 11,050 participants aged ≥20 years were evaluated. Among male patients with CKD, FN BMD was positively related to eGFR, but this correlation was not found in female CKD patients. However, LS BMD did not show any significant association with GFR. In addition, FN BMD showed a positive correlation between osteoporosis and CKD stage; however, LS BMD did not show any positive correlation. These results demonstrate a significant risk of hip fracture in CKD patients	Drawing concrete conclusions is difficult for a cross-sectional study	Patients with CKD had a higher prevalence of hip fracture than spine fracture
Nazzal et al., 2020 [13]	Cross-sectional study	A total of 194 ESRD patients participated in a cross-sectional study in a Palestinian hospital. Due to their ESRD, they were undergoing dialysis (both hemodialysis and peritoneal dialysis) on a regular basis. Among them, 42.8% of patients developed osteoporosis, while 40.2% developed osteopenia. However, patients aged >60 years had a higher percentage of PTH, osteopenia, and osteoporosis	Evaluation of changes in BMD was difficult as it was a cross-sectional study. Moreover, peritoneal dialysis patients were very low in number, so the relationship between dialysis type and MBD could not be identified	ESRD patients had a higher percentage of osteopenia and osteoporosis
Chen et al., 2018 [24]	Longitudinal aging study	A six-year longitudinal aging study was performed among 1,477 participants in Amsterdam. Women had a GFR of >74 mL/minute/1.73 m^2^, while men had a GFR of <57 mL/minute/1.73 m^2^. This early decrease in renal function was associated with a 38% increased incidence of risk of fracture. However, patients with stage 3a and 3b CKD manifested a 28% and 46% increased incidence, respectively. Also, men with early renal dysfunction had lower FN BMD	Renal function was evaluated once and GFR could not be estimated directly	Patients with renal dysfunction were associated with an increased incidence of fracture anywhere in the body
Liu et al., 2019 [23]	N/A	78 patients underwent a study for two years at the Union Hospital. Inflammatory mediators, minerals, hormones, and vitamins were analyzed. IL-6, CRP, TNF-α, serum phosphate, potassium, and blood urea nitrogen were elevated. While serum calcium, BMD, and vitamin D reduced with the progression of the disease. Hence, the severity of the disease exhibits a strong association with osteoporosis. As the disease got more severe, the condition of osteoporosis became worse as well	N/A	Increased severity of CKD is associated with a more severe condition of osteoporosis
Connelly et al., 2018 [4]	Review article	DXA scan is equally useful as a prognostic tool for the normal population as for patients with stage 1–3 CKD. Efficacy of bisphosphonates, SERM, RANKL inhibitors, and PTH agonists were similar for patients with mild-to-moderate CKD, along with patients who underwent renal transplantation. Current treatment guidelines suggest using bisphosphonates and other osteoporosis treatments, even if a mild-to-moderate decline in kidney function is noticed	N/A	DXA scan has shown accuracy when used to measure BMD in patients with stage 1–3 CKD and should be used as a guide for osteoporosis treatment
Yavropoulou et al., 2020 [3]	Case-control study	A total of 30 age- and sex-matched CKD–MBD patients (cases) and 30 healthy individuals (controls) were included in this study. Compared to controls with normal GFR, CKD–MBD patients showed lower serum miRNAs, which has been linked to osteoblasts (miRNA-23a-3p, miRNA-124-3p and miRNA-21-5p). There was a high sensitivity (78%) and specificity (83%) of expressed miRNAs in patients with CKD–MBD and osteoporosis, suggesting that it could be a potential diagnostic biomarker and therapeutic target for such patients	Altered miRNAs expression in the study population could be due to secondary hyperparathyroidism. In addition, the sample size was relatively small, so variations in bone remodeling and calcium metabolism over the study period were not assessed	CKD–MBD is associated with a significantly altered expression of miRNAs linked to osteoblasts and osteoclasts
Hyun et al., 2020 [53]	Observational cohort study	Data from a cohort study in Korea were analyzed to investigate the outcome in CKD patients. A total of 2,238 adults with CKD were enrolled initially. Among them, 2,128 participants underwent BMD measurement via DXA and T-score. Lower BMD was prevalent in CKD patients with higher stage	A causal relationship between low BMD and incident ESRD might be difficult to establish due to the study design and because participants self-reported the health questionnaires. However, DXA was unable to differentiate various renal osteodystrophy	Patients with CKD, who had a lower BMD, also had a greater occurrence of ESRD than those with normal BMD
Damasiewicz et al., 2018 [35]	Review article	The pathophysiology and epidemiology of renal osteodystrophy are discussed. This article also includes how the diagnosis of ROD is made and how advances and proper treatment with various drugs can lower the risk of fracture in CKD patients. A new approach and advancement of treatment are needed to decrease the fracture risk in CKD patients, especially in CKD3-5D. Thus, more research and collaboration with other specialties such as endocrinology will be helpful to overcome this ROD-related fracture	N/A	Patients with CKD have greater than a 10‐fold higher fracture rate than those without CKD
Cohen-Solal et al., 2020 [50]	Review article	The article mainly discusses the pathophysiological effects of biochemical and mineral components implicated in MBD and the danger of fracture in CKD patients. MBD exhibits many skeletal presentations from pain to fragility in these patients. Different diagnostic methods that are used to detect bone fracture are discussed in brief. Therefore, composite approaches including mineral biomarkers and imaging techniques are needed to identify fragility and for treatment options	N/A	As bone fracture is multiplexed in patients with CKD, integrative management is needed
Hsu et al., 2020 [9]	Systematic review and meta-analysis	A total of 178 articles up to 2020 from PubMed and Medline are reviewed in this article. Calcium, phosphate, PTH, vitamin D, FGF, and klotho abnormalities influence bone turnover, mineralization, growth, and strength. DXA or FRAX are used to monitor fracture risk whereas bALP and iPTH help to evaluate bone turnover. Antiresorptive or anabolic agents are mostly used for the treatment. However, vitamin and mineral addition in the diet, physical activity, and smoking termination may help in the prevention	N/A	Calcium, phosphorus, PTH, vitamin D, FGF, and klotho peculiarities help in the pathogenesis of osteoporosis
Palmer et al., 2019 [44]	Review update article	Bisphosphonate can help reduce fracture and bone pain after kidney transplantation. It is not evident if therapy (bisphosphonate) can prevent skeletal complications such as spinal deformity or bone necrosis, especially in patients with a history of a kidney transplant. It is also uncertain if vitamin D has any effect on cardiovascular, death, skeletal, or transplant function outcomes. The impact of bone treatment after kidney transplantation requires further research	N/A	Bisphosphonate therapy could minimize bone pain and fracture after kidney transplantation. Note that the consequence of treatment on BMD is not clear

Limitations

Our study has certain limitations. First, we could not include all the articles published during and after 2011 in our study. Second, our main focus in this systematic review was on analyzing osteoporosis, specifically in patients with CKD. Finally, we excluded cases of osteoporosis related to age, sex, hormonal imbalance, and geographical distribution. Hence, additional complications of CKD were beyond the scope of our discussion. Therefore, further collaborative multidisciplinary studies are needed in this regard.

## Conclusions

We demonstrated a positive correlation between CKD and osteoporosis, specifically, an increased prevalence of osteoporosis was observed in CKD patients. In addition, we discussed how CKD-induced vitamin and mineral homeostasis disruption leads to secondary hyperparathyroidism, subsequent abnormal bone remodeling, and, eventually, osteoporosis and fracture. In light of these findings, understanding the pathogenesis and implementing both invasive and noninvasive diagnostic tools help in early detection and initiating proper management and treatment protocol to halt the progression of CKD-related bone abnormalities. Moreover, vitamin supplementation and lifestyle modifications can assist in osteoporosis prevention. Therefore, additional studies are needed to determine the fundamentally extended pathophysiology and to identify the appropriate drugs and approaches for managing and preventing CKD-induced osteoporosis.
